# Controlled Release of Human Dental Pulp Stem Cell‐Derived Exosomes from Hydrogels Attenuates Temporomandibular Joint Osteoarthritis

**DOI:** 10.1002/adhm.202402923

**Published:** 2024-12-23

**Authors:** Victor Diez‐Guardia, Yajing Tian, Yunzhe Guo, Jiaying Li, Shengjie Cui, Cécile A. Dreiss, Eileen Gentleman, Xuedong Wang

**Affiliations:** ^1^ Centre for Craniofacial and Regenerative Biology King's College London London SE1 9RT UK; ^2^ Department of Orthodontics Peking University School and Hospital of Stomatology Beijing 100081 China; ^3^ National Clinical Research Center for Oral Diseases & National Engineering Laboratory for Digital and Material Technology of Stomatology Beijing 100081 China; ^4^ Beijing Key Laboratory of Digital Stomatology Beijing 100081 China; ^5^ Center of Stomatology China–Japan Friendship Hospital Beijing 100029 China; ^6^ Institute of Pharmaceutical Science King's College London London SE1 9NH UK; ^7^ Department of Biomedical Sciences University of Lausanne Lausanne 1005 Switzerland

**Keywords:** dental pulp stem cells, exosomes, hydrogel, osteoarthritis, temporomandibular joint, tissue repair

## Abstract

Temporomandibular joint osteoarthritis (TMJOA) is a painful inflammatory condition that limits mouth opening. Cell‐derived exosomes, which have anti‐inflammatory effects, are emerging as a treatment for TMJOA. Injection of dental pulp stem cells (DPSCs), which secrete exosomes, can moderate tissue damage in a rat model of TMJOA. However, injected exosomes are quickly cleared, necessitating repeated injections for therapeutic efficacy. Here, vinyl sulfone‐modified hyaluronic acid (HA‐VS) hydrogels, suitable for encapsulating exosomes are formulated. HA‐VS hydrogels degrade in the presence of hyaluronidase and allow for the release of beads of similar size to exosomes over 3 to 6 days. In a rat model of TMJOA, injection of exosomes or exosomes within HA‐VS hydrogels significantly attenuated damage‐mediated subchondral bone loss as determined by micro‐computed tomography, and reduced inflammatory and tissue damage scores as assessed by histology. Overall, DPSCs‐derived exosomes attenuated joint damage, but treatment with exosomes within HA‐VS hydrogels shows additional protective effects on subchondral bone maintenance and integrity. These findings confirm the protective effects of DPSCs‐derived exosomes in moderating tissue damage in TMJOA and suggest that combining exosomes with HA hydrogels can further promote their therapeutic effects.

## Graphical abstract

Exosomes can reduce tissue damage in temporomandibular joint osteoarthritis (TMJOA), but rapid clearance limits their efficacy. This study encapsulates exosomes in hyaluronic acid hydrogels for controlled release. In a rat model, hydrogel‐encapsulated exosomes outperform free exosomes in preserving bone integrity and reducing tissue destruction, highlighting hydrogel‐based systems as a promising strategy for TMJOA therapy.

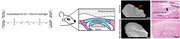

## 1 Introduction

The temporomandibular joint (TMJ), which joins the mandibular condyle of the jaw to the glenoid fossa on the temporal bone, allows for chewing and speaking. Osteoarthritis of the TMJ (TMJOA) is a degenerative disease whereby the cartilaginous surfaces of the TMJ are damaged and the underlying subchondral bone undergoes pathological remodeling, resulting in pain and poor joint function. The drivers of TMJOA are complex, and multifactorial, and remain poorly understood.^[^ ^1^ ^]^ TMJOA is associated with a strong and sustained immune response marked by oxidative stress and secretion of matrix metalloproteinases (MMP), which actively degrade the cartilage.^[^ ^1^, ^2^, ^3^ ^]^ To date, there are no therapies that can prevent or reverse TMJOA progression.^[^ ^4^ ^]^

Dental pulp stem cells (DPSCs) are a mesenchymal cell population that resides in the postnatal dental pulp.^[^ ^5^ ^]^ DPSCs have been used to ameliorate inflammation in a range of conditions, including spinal cord injury and rheumatoid arthritis.^[^ ^6^, ^7^ ^]^ Moreover, in a rat model of TMJOA, injection of DPSCs prevented the progression of TMJOA by attenuating CD4 T cell invasion and inhibiting STAT1 activation, which in turn reduced the secretion of MMP3 and MMP13.^[^ ^8^ ^]^ Indeed, DPSCs‐injected rats showed better maintenance of subchondral bone structure and a partial condylar surface recovery compared to non‐treated controls.^[^ ^8^ ^]^ Nevertheless, DPSCs must be harvested from extracted teeth, effectively precluding their use in autologous therapies,^[^ ^9^, ^10^ ^]^

One of the proposed mechanisms by which DPSCs promote tissue repair is through the secretion of exosomes, which contain lipids, proteins, and miRNAs,^[^ ^11^ ^]^ and have been shown to modulate immune cell responses and promote extracellular matrix (ECM) deposition.^[^ ^12^, ^13^, ^14^ ^]^ Although the precise mechanism by which DPSCs‐derived exosomes mediate tissue repair is still unknown,^[^ ^15^, ^16^ ^]^ therapies based on delivery of allogenic exosomes to treat various diseases including in osteochondral tissues,^[^ ^17^, ^18^ ^]^ offer great promise.^[^ ^19^, ^20^ ^]^ Nevertheless, one of the potential limitations of direct exosome injection is their quick clearance, which in the case of synovial joints like the TMJ, is likely mediated by diffusion through the synovial fluid. Indeed, although some reports describe that exosomes persist in tissues 24 h after injection,^[^ ^21^, ^22^ ^]^ others find that when intravenously administered, they are cleared via hepatic and renal routes after just 6 h.^[^ ^23^ ^]^ Thus, repeated injections may be required for therapeutic efficacy, which is not clinically feasible.

Hydrogels are polymer‐crosslinked networks that absorb several times their weight in water and are promising delivery vehicles in regenerative medicine applications.^[^ ^24^ ^]^ Hydrogels can be designed to have different properties such as degradability, stiffness, and gelation kinetics.^[^ ^25^ ^]^ Among hydrogel materials, hyaluronic acid (HA) is particularly promising for TMJ applications.^[^ ^26^, ^27^ ^]^ HA is a glycosaminoglycan naturally found in human ECMs that is comprised of repeating units of D‐glucuronic acid and N‐acetylglucosamine. HA elicits minimal immune responses and has been shown to promote cell proliferation and survival.^[^ ^28^ ^]^ In cartilage, HA also serves as a lubricant and nutrition source in the joint synovial fluid.^[^ ^29^ ^]^ As it is naturally targeted by hyaluronidases, which are ubiquitous in tissues, HA is also degradable in vivo. This contrasts with other polysaccharides used to form hydrogels, such as alginate. Moreover, injection of pure sodium hyaluronate has been reported to successfully treat pain symptoms^[^ ^30^ ^]^ and joint crepitus^[^ ^31^ ^]^ in TMJOA patients. HA is also readily available to GMP standards suitable for use in humans.

Here, we hypothesized that exosomes encapsulated within a hydrogel that allows for their retention and sustained release could be applied using a single minimally invasive procedure to moderate joint damage. To test this, we created HA‐based hydrogels^[^ ^25^ ^]^ and investigated their potential to release DPSCs‐derived exosomes and impact tissue repair in a rat model of TMJOA. We demonstrate that HA hydrogels can retain particles of similar sizes to exosomes and release them over time and that when used in conjunction with exosomes derived from DPSCs, can attenuate OA‐related cartilage and bone degeneration. Our findings show the promise of combining slow‐release biomaterials with exosomes to modulate tissue repair in the TMJ and highlight a clinically feasible method for the delivery of a potential therapeutic agent.

## 2 Materials and Methods

### 2.1 Siliconization of Glass Cylinders

Glass cylinders (SciQuip, 6 mm x 8 mm) were treated with Sigmacote (Sigma‐Aldrich) for 30 min, according to the manufacturer's instructions. Treated cylinders were air‐dried for 24 h, rinsed with deionized water (dH_2_O), and autoclaved prior to experiments.

### 2.2 Synthesis of Vinyl Sulfone‐Modified Hyaluronic Acid (HA‐VS)

HA‐VS was synthesized as in a previously established protocol.^[^ ^32^ ^]^ Briefly, 200 mg of HA (100–150 kDa, Lifecore Biomedical) was dissolved in 20 mL of 0.1 mm NaOH in dH_2_O. Subsequently, 568 µL of pure divinyl sulfone (DiVS) (Sigma‐Aldrich) was added at a molar ratio of 16:1 (DiVS: repeating unit of HA). The mixture was gently shaken at 160 rpm for 30 min at room temperature (RT), and the reaction was quenched by adding 600 µL of 0.5 m HCl. The resulting product was purified using dialysis against dH_2_O for 48 h at RT, changing the water every 24 h, followed by lyophilization. The material was then treated with 540 Gy gamma irradiation and stored at ‐20 °C prior to use.

### 2.3 Characterization of HA‐VS

The degree of modification of HA‐VS was determined using proton NMR. Briefly, 5 mg of HA‐VS was dissolved in 600 µL of deuterium oxide and then analyzed on a 400 MHz Bruker instrument. The degree of modification (DOM) was defined as the fraction of repeating units of HA that were modified with VS. Protons from VS groups produce signal peaks at ≈6.3 and ≈6.9 ppm, while protons from methyl groups have a peak at ≈2 ppm. By integrating these peaks, the DOM was determined using the following equation:

(1) $$\mathrm{DOM}=\frac{\mathrm{Integration}\;\mathrm{of}\;\mathrm{Pea}k_{\mathrm{VS}}}{3}/\frac{\mathrm{Integration}\;\mathrm{of}\;\mathrm{Pea}k_{\mathrm{Methyl}}}{3}$$

### 2.4 Preparation of HA‐VS Hydrogels

HA‐VS and dithiol polyethylene glycol (PEG‐SH) (1000 Da) (JenKem) were dissolved in Dulbecco's Modified Eagle Medium (DMEM) (Gibco) and Dulbecco's Phosphate‐Buffered Saline (DPBS) (Gibco), achieving final concentrations of 0.04 and 0.107 mg µL^−1^, respectively. The PEG‐SH solution was then passed through a 0.22 µm filter (Millex). To create hydrogels, components were combined as outlined in **Table** **1** and allowed to crosslink in Sigmacote‐treated glass cylinders within 48 well plates for 30 min at 37 °C before glass cylinders were removed with sterile forceps.

**Table 1:** Compositions of 1% and 2% HA‐VS hydrogels.

Gel type	Mixture of DMEM and DPBS (3:1, v/v)	HA‐VS solution [0.04 mg µL^−1^]	Di‐thiol PEG solution [0.107 mg µL^−1^]
1% HA‐VS hydrogel	30 µL	12.5 µL	2.5 µL
2% HA‐VS hydrogel	15 µL	25 µL	5 µL

### 2.5 Hydrogel Degradation and Fluorescent Bead Release

Hydrogels were placed in 1 mL of a freshly prepared solution of 10 U mL^−1^ hyaluronidase from bovine testis (HAase, Sigma) in DPBS or in DPBS alone (control) and maintained at 37 °C. HAase+DPBS and DPBS solutions were refreshed every day and the supernatant was stored at −20 °C for subsequent analysis.

Quantification of D‐glucuronic acid, one of the repeating sugar units that comprises HA, was performed using a carbazole assay, as previously described.^[^ ^33^ ^]^ Briefly, 50 µL of hydrogel supernatant was combined with 200 µL of 25 mm sodium tetraborate decahydrate in sulfuric acid. The mixture was heated to 99 °C for 15 min, and then cooled to 4 °C. 8 µL of solution containing 0.125% (w/v) carbazole (Sigma‐Aldrich) in ethanol was then added, and the solution heated again to 99 °C for 15 min, and then cooled to 4 °C. The resulting solution was then transferred to a 96‐well plate and absorbance read at 495 nm using a colorimetric plate reader (CLARIOstar, BMG Labtech). The concentration of D‐glucuronic acid was then determined using a standard curve generated by a series of known D‐glucuronic acid solutions.

To simulate exosome release, 1 mg mL^−1^ of 100 or 200 nm fluorescent beads (PS‐COOH, 365 nm/610 nm, Bangs Laboratories, Inc) were incorporated into hydrogels during the crosslinking process. Hydrogels were then incubated in the presence and absence of HAase (as above), and supernatants were collected. Supernatants were measured on a fluorescence plate reader (365 nm/610 nm, excitation/emission; CLARIOstar, BMG Labtech), and the percent of the total beads released was calculated based on a standard curve established using a series of solutions containing known concentrations of fluorescent beads.

### 2.6 Rheological Measurements

Dynamic time sweep measurements were carried out on a DHR3 rheometer (TA Instruments). 50 µL of solution, creating 1% or 2% HA‐VS hydrogels (Table 1), was prepared fresh, and cast on an 8 mm diameter parallel plate geometry within ≈20 s. To prevent hydrogel evaporation, a thin layer of paraffin oil was applied around the sample, and the gap between the plates was set to 1 mm. Strain amplitude was set within the linear viscoelastic regime. Dynamic time sweep measurements were taken every 10 s at a temperature of 37 °C, which was controlled by a Peltier unit. The gelation point was determined as the crossover between G′ and G″, adding the time for loading the sample (+≈20s). The plateau modulus was determined by taking the mean G’ over the last 5 minutes of the measurement.

### 2.7 Isolation and Culture of Human DPSCs

Patients gave informed consent, and ethical approval for the study was provided by the Peking University School of Stomatology Institutional Review Board (PKUSSIRB‐201311103). Third molars were obtained from individuals aged 18 to 25 years undergoing routine extraction procedures. Under aseptic conditions, dental pulp tissues were removed from molars and minced into small pieces. 3 mg mL^−1^ collagenase and 4 mg mL^−1^ dispase (both from Solarbio) were mixed in a 1:1 ratio in DPBS, added to tissue pieces, and incubated for 60 min at 37 °C. The resulting solution was pelleted by centrifugation, and human DPSCs were collected and cultured in alpha minimum essential medium (αMEM, Gibco) supplemented with 10% fetal bovine serum (FBS, Gibco) and 1% penicillin/streptomycin (Gibco) under standard conditions (37 °C; 5% CO_2_/95% air). Upon reaching 80% confluence, cells were trypsinized and passaged at a ratio of 1:4. Cells from the 4th and 5th passages were used in experiments.

### 2.8 Isolation and Characterization of Exosomes

When human DPSCs reached 80% confluence, the standard culture medium was replaced with αMEM supplemented 1% penicillin/streptomycin, which was collected after 24 h. The supernatant was then centrifuged at 300, 2000, and 10 000×*g* for 10 min each to remove cellular debris. The remaining supernatant was then twice subjected to centrifugation at 100 000×*g* for 70 min to isolate exosomes. Exosomes were resuspended in cold DPBS to prevent aggregation, and their concentration was determined by BCA protein assay (Solarbio, China, following the manufacturer's instructions). The concentration of exosomes was then adjusted to 3 g L^−1^ for experiments. Exosomes in DPBS were stored at 4 °C for no longer than 12 h before injection.

Exosome morphology and size were characterized following a previously described protocol.^[^ ^34^ ^]^ In short, for imaging, purified exosomes were fixed in 2.5% glutaraldehyde in 0.1 m sodium cacodylate buffer (pH 7.0) for 1 h at 4 °C. Exosomes were then rinsed three times with 0.1 m sodium cacodylate buffer at RT, 10 min per rinse. Exosomes were then post‐fixed with 2% osmium tetroxide in dH_2_O for 1 h at 4 °C. Exosomes were rinsed 3 more times with 0.1 m sodium cacodylate buffer at RT, and then placed in increasing concentrations of acetone (50%, 60%, 70%, 80%, 90%, 95%, and 100%) for 10 min each at 4 °C. Next, exosomes were incubated in 3:1, 1:1, 1:3 solutions of acetone: Epon 812 embedding mixture for 30 min each, and finally overnight in 100% Epon 812 at RT. Exosomes were then cast in a mold and baked for 24 h at 65 °C in Epon 812. 60 nm sections were prepared using an ultra‐microtome (Leica EM UC7, Germany) and double stained with 2% uranyl acetate for 20 min and Reynolds lead solution (1.33 g lead nitrate and 1.76 g sodium citrate in 50 mL dH_2_O) for 10 min at RT. Images were captured using transmission electron microscopy (TEM; JEM‐1400, JOEL, Japan) at 80 kV. Nanoparticle tracking analysis (NTA; NanoSight NS300 Technology, Malvern, UK) was carried out on 10^6^–10^9^ particles/mL and analyzed using NanoSight NTA v3.40, following the manufacturer's instructions.

### 2.9 Animal Experiments

The animal procedures were approved by the Peking University Animal Ethics Committee prior to the initiation of the study (approval number: LA2021288) and performed in accordance with the approved guidelines. Twenty‐eight female Sprague‐Dawley rats (8 weeks old) with a mean weight 204.4 g (range 195–220 g) were used in this study. Rats were randomly divided into 4 groups (*n* = 7) hereafter referred to as: Control, HA‐VS hydrogel, Exosome, and HA‐VS+exosome. All rats (a total of 56 TMJs) received intra‐articular injections of 50 µL monosodium iodoacetate (MIA) in DPBS (10 ug µL^−1^) to induce the TMJOA model. One week after TMJOA induction, rats received intra‐articular injections of either 50 µL DPBS (control), 50 µL HA‐VS hydrogel solution (HA‐VS hydrogel), 50 µL exosome solution dissolved in DPBS (1 ug µL^−1^) (Exosome), or 50 µL exosome solution dissolved in the HA‐VS hydrogel solution (HA‐VS+exosome; 1 ug µL^−1^ of exosomes). One and 2 weeks after injection, three and four rats from each group, respectively, were euthanized, and treated TMJs were surgically removed for analysis.

### 2.10 Micro‐Computed Tomography

TMJs were immersed in 10% (v/v) formalin for overnight fixation and analyzed using a micro‐computed tomography (microCT) system (SkyScan1172, Bruker, Germany). Samples were scanned coronally at 55 kV and 860 µA with a 12.5 µm‐effective pixel size and reconstructed with matching software (CTAn and CTVol). Images were rotated until the condyle at the most anterior point and the most posterior point were aligned within the horizontal plane, and a 1.3mm × 1.3mm × 0.3 mm volume of interest was selected (mid‐point between the most anterior and the tallest part of the condyle). Parameters, including the percentage of bone volume over total volume (BV/TV, %), the ratio of bone surface to bone volume (BS/BV, 1 mm^−1^), trabecular separation (Tb.Sp, mm), and trabecular number (Tb·N, 1 mm^−1^) were automatically calculated using the instrument's software.

### 2.11 Histological Examination and Analysis

Fixed TMJs were decalcified in 10% (w/v) EDTA (Servicebio) in dH_2_O (pH 7.2) at 25 °C for 4 weeks, embedded in paraffin, sectioned and stained with hematoxylin and eosin. Histological scoring was carried out by 2 experienced researchers who were blinded to the groups and assigned an agreed single score per image, according to the modified Mankin scoring system and inflammation criteria.^[^ ^8^, ^35^, ^36^, ^37^ ^]^

### 2.12 Statistical Analysis

In vitro data are presented as mean ± standard deviations. Statistical analyses were carried out using GraphPad Prism software (v 10.1.0). In vitro comparisons were carried out using a multiple unpaired t‐test (two‐stage step‐up using the Benjamini, Krieger, and Yekutieli method). MicroCT data and Mankin scores were analyzed by ANOVA using a post‐hoc Tukey test. Comparisons were considered significant if *p* < 0.05.

## 3 Results

**Figure 1:**
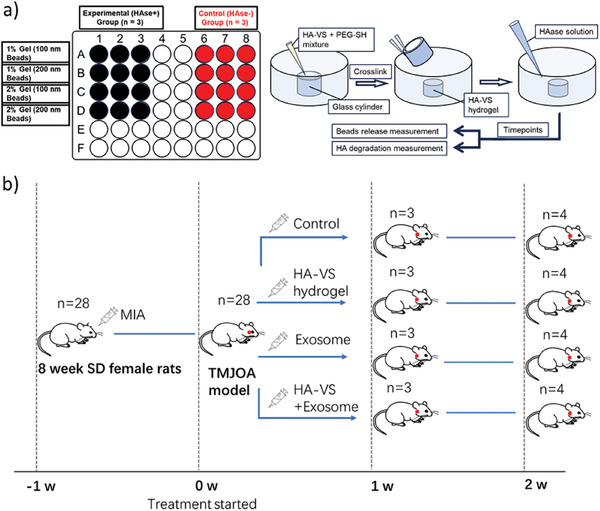
Schematic illustrating the experimental design. a) In vitro study in which 1% and 2% HA‐VS hydrogels were tested for D‐glucuronic acid and fluorescent bead release in the presence and absence of hyaluronidase (HAase). b) In vivo study in which a TMJ damage model was established (MIA) and rats (*n* = 7 per group) treated with either DPBS, HA‐VS hydrogel, exosomes, or HA‐VS containing exosomes for up to two weeks. 3 rats per group were examined at 1 week post‐treatment, and 4 rats per group at 2 weeks post‐treatment.

To create a system suitable for delivering exosomes to the TMJ, we designed a series of in vitro and in vivo experiments (**Figure** **1**). The in vitro experiments aimed to optimize an HA‐based hydrogel formulation to degrade and release particles of similar size to exosomes over time. The in vivo study was designed to assess the ability of the hydrogel, exosomes, and the hydrogel combined with exosomes to attenuate the degradation of joint tissues in a rat model of chemically induced TMJOA.

### 3.1 HA‐VS Polymer Concentration Impacts Hydrogel Gelation Kinetics and Stiffness

**Figure 2:**
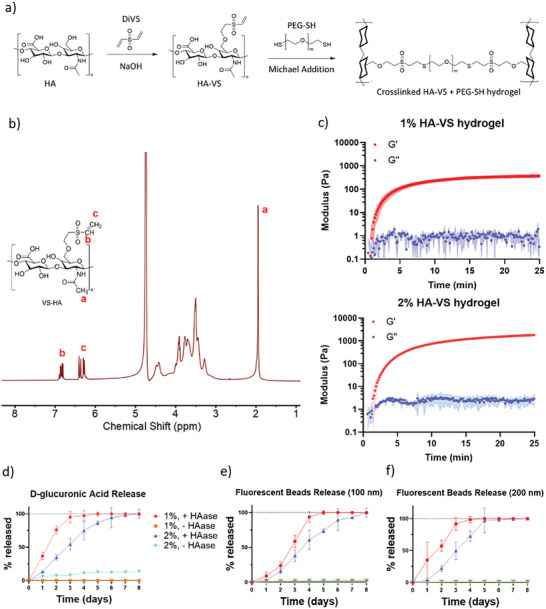
Altering polymer concentration impacts gelation kinetics, mechanical properties, and degradation rates of HA‐VS hydrogels. a) Strategy to chemically modify HA with VS groups to produce hydrogels using a Michael addition. b) Proton NMR spectrum of VS‐modified HA (“a” 3H from HA methyl group, “b” 1H from vinyl group, “c” 2H from vinyl group). c) Mean dynamic time sweep measurement of 1% and 2% HA‐VS hydrogels (*n* = 3, shaded area shows standard deviation). d) Release of D‐glucuronic acid from 1% and 2% HA‐VS hydrogels in the presence and absence of hyaluronidase (HAase) over time (*n* = 3 per time point). e) Release of 100 and f) 200 nm diameter fluorescent beads from 1% and 2% HA‐VS hydrogels in the presence and absence of hyaluronidase (HAase) over time (*n* = 3 per time point).

As our goal was to create a material suitable for injection in the TMJ, we first aimed to create hydrogels that would both crosslink via a Michael addition in a time frame suitable for injection into the TMJ and would safely degrade over time, releasing the exosomes. To achieve this, we first modified HA with vinyl sulfone (VS) groups to allow for crosslinking in the presence of PEG‐SH (**Figure** **2**a). ^1^H NMR analysis confirmed successful modification of the HA, with a degree of modification of 48% (Figure 2b). We next aimed to characterize hydrogels to determine if modulating HA polymer concentration impacted hydrogel gelation kinetics and degradation properties. We prepared 1% and 2% (w/v) HA‐VS hydrogel solutions and crosslinked them with PEG‐SH using a 1:1 molar ratio of VS: SH groups. Dynamic time sweep measurements with strain amplitude set within the linear viscoelastic regime (Figure S1, Supporting Information) identified a cross‐over point between G′ and G″ after less than 2 minutes. G’ of both formulations then increased quickly and achieved plateaus at ≈375 Pa for the 1% HA‐VS formulation and ≈1870 Pa for the 2% HA‐VS formulation (Figure 2c). Surgically delivering a hydrogel into the TMJ requires balancing handling, such that the material does not crosslink prior to injection, with quick gelation in situ to preclude dilution. Our finding that HA‐VS hydrogels crosslinked within minutes should make them suitable for applications in the TMJ.

### 3.2 HA‐VS Polymer Concentration Affects Hydrogel Degradation and Release of Exosome‐Sized Particles

We next aimed to evaluate the degradation of HA‐VS hydrogels by measuring the release of D‐glucuronic acid (one of the repeating sugar units that make up HA) in solutions with and without the enzyme hyaluronidase (HAase), which is ubiquitous in mammalian tissues and degrades HA. As expected, increasing hydrogel polymer concentration slowed the degradation of HA‐VS hydrogels. Indeed, doubling the polymer concentration of HA‐VS from 1% to 2% extended the time required for full degradation from 3 days (*p* > 0.1, compared to 100% degradation) to 6 days (*p* > 0.5, compared to 100% degradation) (Figure 2d). Importantly, both 1% and 2% HA‐VS formulations were resistant to spontaneous water hydrolysis in solution over 8 days.

We then set out to assess the ability of the hydrogels to control the release of particles of similar size to exosomes. Exosomes have been reported to have diameters in the range of 30 to 150 nm,^[^ ^11^, ^38^ ^]^ and our analyses of DPSC‐derived exosome size identified a mean diameter of 120 nm (see below and Figure S2, Supporting Information). Therefore, we loaded 1% and 2% HA‐VS formulations with fluorescent beads that were either 100 or 200 nm in diameter and tested the release of beads over time in the presence and absence of HAase using a fluorescence plate reader. The pattern of bead release followed a similar trend to that of polymer degradation, as bead release was quicker in the 1% compared to the 2% HA‐VS formulation. For 100 nm beads, bead release was no different in 1% and 2% hydrogels after 2 days in culture (*p* > 0.1) (Figure 2e). However, after 3 days in culture, bead release was significantly higher in 1% hydrogels compared to 2% (*p* < 0.05), and by day 4, 1% hydrogels had fully released their beads (*p* > 0.1, compared to 100% bead release). In contrast, full bead release was not achieved in 2% hydrogels until day 8 (*p* > 0.5, compared to 100% bead release).

Reflecting trends observed with 100 nm fluorescent beads, the release of 200 nm beads was faster in 1% compared to 2% hydrogels (Figure 2f). However, 200 nm bead release was slightly quicker, as bead release was significantly higher in 1% hydrogels compared to 2% at day 2 (*p* < 0.01) and full bead release achieved by day 3 (*p* > 0.1, compared to 100% bead release). Similarly, in 2% hydrogels, full release of 200 nm beads was achieved by day 5 (*p* > 0.5 compared to 100% bead release). These data suggest that lower polymer concentration hydrogels offer faster release of exosome‐sized particles and that larger particles are released faster, perhaps because larger particles may disrupt hydrogel gelation, making it more susceptible to degradation. Moreover, release profiles of fluorescent beads reflected what would be expected based on the hydrogel degradation kinetics, providing confidence that bead release could be used as an indicator of exosome release. As observed in degradation experiments, only minimal bead release was evident from hydrogels not exposed to HAase, suggesting that release of exosome‐sized particles from HA‐VS hydrogels is controlled by HAase activity.

### 3.3 hDPSCs Exosomes Attenuate Subchondral Bone Deterioration in a Model of Progressive TMJOA

Next, to determine if exosomes and exosomes released from HA‐VS hydrogels could attenuate joint damage in vivo, we induced TMJOA by injecting MIA into both TMJs of a rat model. MIA is an inhibitor of glyceraldehyde‐3‐phosphate dehydrogenase and is used in OA models of the TMJ where it causes cartilage degeneration and subchondral bone remodeling.^[^ ^39^ ^]^ We then derived exosomes from human DPSCs cultures and assessed their size using a combination of nanoparticle tracking analysis and transmission electron microscopy. These analyses showed that human DPSC‐derived exosomes had diameters of ≈120 nm (Figure S2, Supporting Information). 1 week after MIA‐mediated injury, one of the two injured TMJs were injected with either DPBS (control), HA‐VS hydrogel alone, exosomes alone or HA‐VS hydrogel with encapsulated exosomes (HA‐VS+exosome). Animals were monitored post‐injection, and no animal in any group showed signs of distress or serious inflammation.

**Figure 3:**
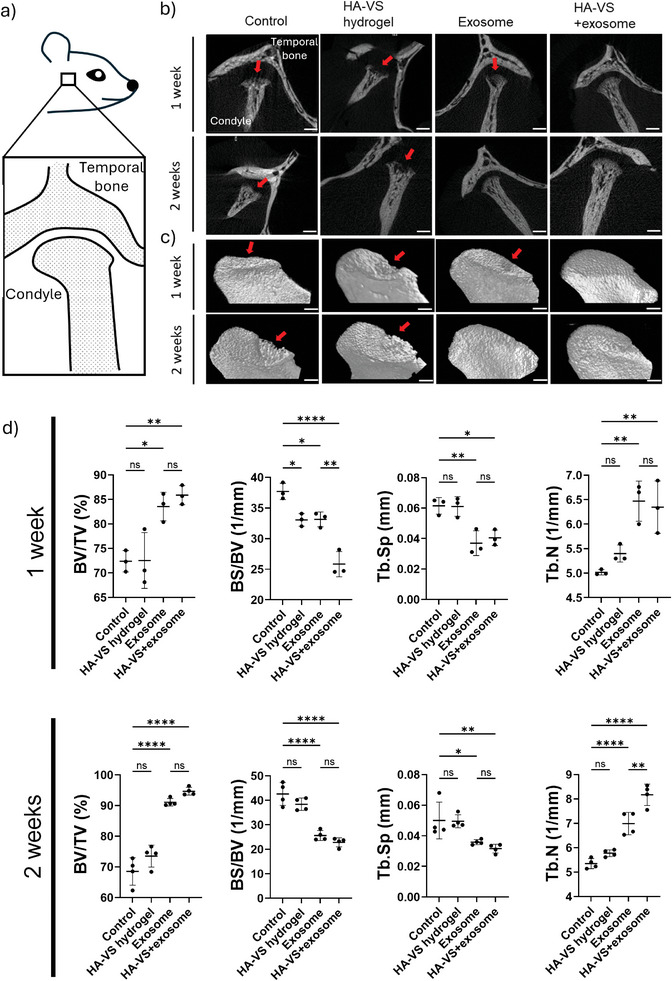
hDPSCs exosomes attenuate subchondral bone deterioration and degenerative changes in progressive TMJOA. a) Diagram depicting sagittal view of the TMJ in a rat. b) Sagittal view and c) 3D reconstructions of TMJ and condyles. Red arrows indicate areas of bone destruction (scale bars of sagittal views = 2 mm; scale bars of 3D reconstructions = 800 µm). d) MicroCT analysis of the percentage of bone volume over total volume (BV/TV, %), the ratio of bone surface to bone volume (BS/BV, 1/mm), trabecular separation (Tb. Sp, mm), and trabecular number (Tb·N, 1 mm^−1^). ANOVA followed by Tukey test; *p < 0.05, **p < 0.01, ***p < 0.001, ****p < 0.0001.

After 1 (*n* = 3) and 2 weeks (*n* = 4), animals were sacrificed, and subchondral bone quality was assessed by microCT (**Figure** **3**a). MicroCT images showed clear deterioration of the subchondral bone in both the control and HA‐VS hydrogel groups at both 1 and 2 weeks post‐injection, with more pronounced joint damage evident at 2 weeks, particularly along the anterior slope of the condyle (Figure 3b,c). Conversely, TMJs that received injections containing exosomes (both exosome and HA‐VS+exosome groups) showed better maintenance of joint structure, particularly of the subchondral bone. At the 2‐week time point, the condylar surfaces of exosome and HA‐VS+exosome groups also seemed more continuous and smoother.

To quantify changes in the subchondral bone in microCT images, we selected a region of interest on the anterior slope of the condyle. 1‐week post‐treatment, all variables including bone volume/total volume (BV/TV), bone surface/bone volume (BS/BV), trabecular spacing (Tb.Sp), and trabecular number (Tb.N) showed significantly better bone maintenance in both exosome‐ and HA‐VS+exosome‐treated groups compared to controls (Figure 3d). Indeed, after 1 week, BV/TV and Tb.N in the exosome only‐treated group were both significantly higher (*p* < 0.05, *p* < 0.01), and BS/BV and Tb.Sp were both significantly lower (*p* < 0.05, *p* < 0.01) compared to controls, with similar significant trends observed after 2 weeks. The HA‐VS+exosome group proved even more protective of bone deterioration, as BV/TV and Tb.N were both significantly higher (*p* < 0.01, *p* < 0.01), and BS/BV and Tb.Sp both significantly lower (*p* < 0.0001, *p* < 0.05) after 1 week of culture compared to controls, which similarly held after 2 weeks. Moreover, after 1 week, BS/BV was significantly lower in the HA‐VS+exosome group (*p* < 0.01), and after 2 weeks, Tb.N was significantly higher (*p* < 0.01) compared to the exosome‐only group. We only observed minimal differences in microCT parameters between the control group and the HA‐VS hydrogel only group, suggesting that HA‐VS hydrogels alone did not have a detrimental effect on bone parameters in the MIA‐induced OA model and that the therapeutic effect in ameliorating bone deterioration was mediated through the exosomes, not the material itself.

### 3.4 hDPSCs Exosomes Prevent Degenerative Joint Changes and Reduce Markers of Inflammation In Progressive TMJOA

**Figure 4:**
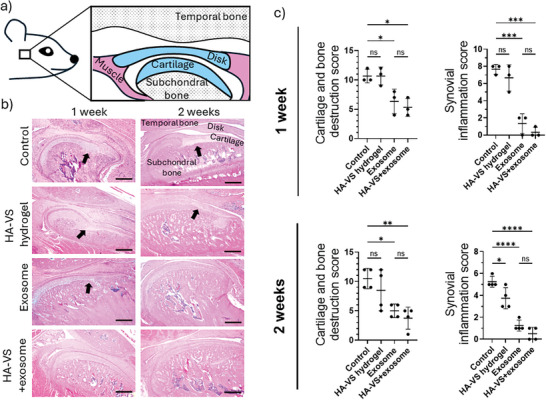
a) Diagram highlighting structures of the rat TMJ. b) Representative images of hematoxylin‐eosin (HE) staining of the TMJ. Compared with the control group and HA‐VS hydrogel group, the inflammatory cell infiltration was minimal and the bone destruction less apparent in joints treated with exosomes or HA‐VS+exosomes. Black arrows indicate areas of cartilage and bone destruction (scale bars = 500 µm). c) Histological scoring of tissue samples for cartilage and bone destruction and synovial inflammation after 1 and 2 weeks (*n* = 3 per group for 1 week, *n* = 4 per group for 2 weeks). ANOVA followed by Tukey test; *p < 0.05, **p < 0.01, ***p < 0.001, ****p < 0.0001.

As microCT had suggested that both treatments with exosomes and HA‐VS+exosomes could limit bone destruction in an in vivo model of TMJOA, we next aimed to assess their impact on soft tissues in the joint by histology (**Figure** **4**a). To accomplish this, we performed standard staining and had two blinded independent researchers evaluate the level of joint degradation and inflammation following a standard Mankin scoring system. Visual examination of the tissue revealed apparent thickening of subsynovial connective tissue and the presence of extensive liquefied necrotic mononuclear cells in both the control group and the HA‐VS hydrogel group (Figure 4b). In addition to synovial inflammation, the loss of peripheral chondrocytes and subchondral bone erosion resulted in significantly higher scores for cartilage and bone destruction and synovial inflammation at both 1 week (*p* < 0.05, for cartilage destruction and *p* < 0.001 for synovial inflammation scores, compared to exosome and HA‐VS+exosome groups in both cases) and 2 weeks post‐treatment (*p* < 0.05 and *p* < 0.01, for cartilage destruction, and *p* < 0.0001 for synovial inflammation scores, compared to exosome and HA‐VS+exosome groups) (Figure 4c). There were no significant differences in scores between the exosome‐only group and the HA‐VS+exosome group for any comparisons; however, HA‐VS hydrogel alone resulted in a significantly decreased synovial inflammation score compared to the control (*p* < 0.05) at 2 weeks post‐treatment.

## 4 Discussion

Here, we investigated the use of HA‐based hydrogels for the controlled release of DPSCs‐derived exosomes to mediate repair of damaged joint surfaces in a rat model of TMJOA. Our previous work showed the promise of using DPSCs to treat TMJOA in rats,^[^ ^8^ ^]^ but using cells as a therapy can be costly and impractical, particularly as autologous DPSCs are not readily available. DPSCs’ therapeutic effects are thought to be mediated, at least in part, by their exosomes, but injecting exosomes directly into the body can result in their dilution in extracellular fluids. Because of this, repeated injections may be required to attain a therapeutic effect, which is not clinically feasible.

We aimed to both harness DPSC‐derived exosomes therapeutic potential and avoid their rapid clearance. Our study makes use of HA hydrogels with slow degradation properties to maintain exosomes at the site of injection over ≈1 week. We show that increasing hydrogel polymer concentration both slowed the degradation of hydrogels and the release of exosome‐sized particles. Similar strategies have been used to deliver sclerostin (SOST, a canonical Wnt inhibitor) in in vivo models of TMJ damage. Here, the authors took advantage of the non‐Newtonian viscoelastic properties of high molecular weight HA to deliver SOST without chemically modifying the HA. Their results show that high molecular weight HA can release SOST over a period of ≈40 days and prevent TMJOA progression better than direct SOST injection in rat, pig, and rabbit models.^[^ ^40^ ^]^ While we similarly found that HA could extend the release of fluorescent beads, our in vitro results showed that hydrogels were completely degraded in days, while the high MW HA released SOST over weeks. However, as our in vitro experiments were performed in the presence of exogenous HAase, which is present in the synovial fluid but at unknown concentrations, it is difficult to compare release rates.^[^ ^41^ ^]^ Indeed, tailoring material degradation rates using in vitro approaches will invariably result in limitations as it is extremely challenging to mimic the dynamic in vivo environment where factors such as inflammation‐derived oxidative stress can also influence enzyme production.^[^ ^42^ ^]^

Our approach used a hydrogel to retain exosomes at the site of injection. However, an alternative and effective strategy is to instead target exosomes to areas where they are required by conjugating them with ligands that are specific to cell membrane receptors.^[^ ^43^ ^]^ Such approaches are easily achievable using click reactions;^[^ ^44^ ^]^ however, can suffer from the drawback that exosome cargo, such as miRNAs, may degrade during the conjugation process. This strategy has previously been applied to target cartilage with OA‐protective exosomes. Here, the authors engineered a plasmid that endowed transfected cell‐produced exosomes with an extra membrane protein that preferentially targeted osteoarthritic chondrocytes. Their findings showed the superiority of targeted versus non‐targeted exosomes in reducing the expression of catabolic enzymes such as MMP‐13 and ADAMTS‐5, which play well‐described roles in cartilage destruction,^[^ ^45^, ^46^ ^]^ and protect the cartilage against degradation.^[^ ^47^ ^]^ Nevertheless, while promising, this approach required repeated intra‐articular injections, suggesting that synovial clearance remains an issue for modified exosomes. This contrasts with our hydrogel‐based approach, which only required a single injection. Another alternative to delivering exosomes is to deliver synthetic microspheres containing molecules that might reduce inflammation,^[^ ^48^, ^49^, ^50^ ^]^ or induce tissue healing.^[^ ^51^, ^52^ ^]^ Such simpler approaches may avoid issues such as cargo degradation. However, as the mechanisms by which exosomes mediate therapeutic effects have not been fully elucidated, in many cases, it remains necessary to use cell‐derived components, which contain a richer mixture of biomolecules.^[^ ^16^ ^]^

In addition to studying the impact of DPSCs‐derived exosomes in our rat model of TMJOA, we also assessed the impact of injecting joints with HA‐VS hydrogels alone. The injection of pure HA into the TMJ has been widely reported in clinical practice due to its natural lubricating function.^[^ ^29^ ^]^ Although its effectiveness as a treatment is controversial, it is widely considered to be safe.^[^ ^53^ ^]^ In our study, we used HA modified with VS groups, which to our knowledge, has not been previously tested in the TMJ. Nevertheless, we found that injection of HA‐VS hydrogels alone did not lead to outcomes that were different from injection of DPBS alone. This suggests that the material did not have a detrimental effect on the joint, but contrasts with reports which suggest that unmodified HA can have a therapeutic effect. Thus, further studies with larger numbers of animals, HA with or without chemical modifications, and perhaps different experimental models of TMJOA may be required to determine the efficacy of HA injection as a therapy to treat OA.

Compared to naked exosome injection, we found that injecting exosomes loaded within HA‐VS hydrogels resulted in less TMJ degradation as shown by a significant decrease in BS/BV after 1 week and a significant increase of Tb.N after 2 weeks. BS/BV is a measure of bone surface density, and Tb.N quantifies how many trabeculae are present in a given section of bone. Thus, both Tb.N and BS/BV can often be indicators of bone strength. This is because an increase in BS/BV might suggest thinning of trabeculae or increased bone porosity. Similarly, a decrease in Tb.N might suggest a more sparse trabecular network. Our previous work suggested that DPSCs injected into the TMJ moderate inflammation, at least in part, by moderating MMP activity.^[^ ^8^ ^]^ Thus, our finding that BS/BV was decreased and Tb.N was increased in the HA‐VS+exosome group may suggest that this effect could have been mediated by the prolonged release of exosomes. Such a sustained release could have limited MMP activity in the tissue over a longer period of time compared to exosomes alone, and thus limited bone remodeling, impacting BS/BV and Tb.N. However, further studies would be required to mechanistically link prolonged exosome release to potential anti‐inflammatory and/or bone remodeling effects.

Additionally, histological scores also suggested that injection of exosomes combined with HA‐VS hydrogels provided some protection against degradation and inflammation compared to treatment with exosomes alone, although this effect was not significant. Thus, delivery within HA‐VS hydrogels may enhance the benefits of exosome delivery, but there is still potential room for improvement in our approach as most measures of joint degradation were no different between the two groups. For example, crosslinking hydrogels prior to injection,^[^ ^54^ ^]^ adding adhesive moieties that boost hydrogel‐tissue integration,^[^ ^55^ ^]^ or chemically modifying the HA further to limit degradation are all strategies that may increase exosome retention and provide a more sustained delivery to the injured tissue.

In conclusion, we created HA‐based hydrogels suitable for the delivery of exosomes to the TMJ. We also provide evidence of the safety of our approach and show promising findings that delivery of exosomes from a hydrogel can protect the TMJ from deterioration in a chemically induced model of TMJOA better than injection of exosomes alone. Nevertheless, our findings of the benefits of the HA‐VS hydrogel were subtle and pointed toward a need for further modifications to the material to optimize exosome delivery kinetics.^[^ ^56^ ^]^ Future studies should also consider longer follow‐ups in animal models or less acute models that might better reflect the course of the human disease.

## Conflicts of interest

The authors declare no conflict of interest.

## Supporting information



Supporting Information

## Data Availability

The data that support the findings of this study are available from the corresponding author upon reasonable request.
