# Supplementary material for: Controlled Release of Human Dental Pulp Stem Cell‐Derived Exosomes from Hydrogels Attenuates Temporomandibular Joint Osteoarthritis
Source: Adv Healthc Mater. 2024 Dec 23;14(31):2402923. doi: 10.1002/adhm.202402923 (PMC12683231; doi:10.1002/adhm.202402923)
Supplement: Supplementary file 1 — Supporting Information [file ADHM-14-0-s001.docx]

**Controlled release of human dental pulp stem cell-derived exosomes from hydrogels attenuates temporomandibular joint osteoarthritis**

Victor Diez-Guardia^1^*, Yajing Tian^2,3,4^*, Yunzhe Guo^1^*, Jiaying Li^1^, Shengjie Cui^2,3,4^, Cecile A. Dreiss^5^, Eileen Gentleman^1,6^‡, Xuedong Wang^2,3,4^‡

1. Centre for Craniofacial and Regenerative Biology, King’s College London, London SE1 9RT, UK
2. Department of Orthodontics, Peking University School and Hospital of Stomatology, Beijing, China
3. National Clinical Research Center for Oral Diseases & National Engineering Laboratory for Digital and Material Technology of Stomatology, Beijing, China
4. Beijing Key Laboratory of Digital Stomatology, Beijing, China
5. Institute of Pharmaceutical Science, King’s College London, London SE1 9NH, UK
6. Department of Biomedical Sciences, University of Lausanne, Lausanne 1005, Switzerland

*These authors contributed equally.

‡To whom correspondence should be addressed: [eileen.gentleman@kcl.ac.uk](mailto:eileen.gentleman@kcl.ac.uk) or [wangxuedong@bjmu.edu.cn](mailto:wangxuedong@bjmu.edu.cn)

Supplementary materials


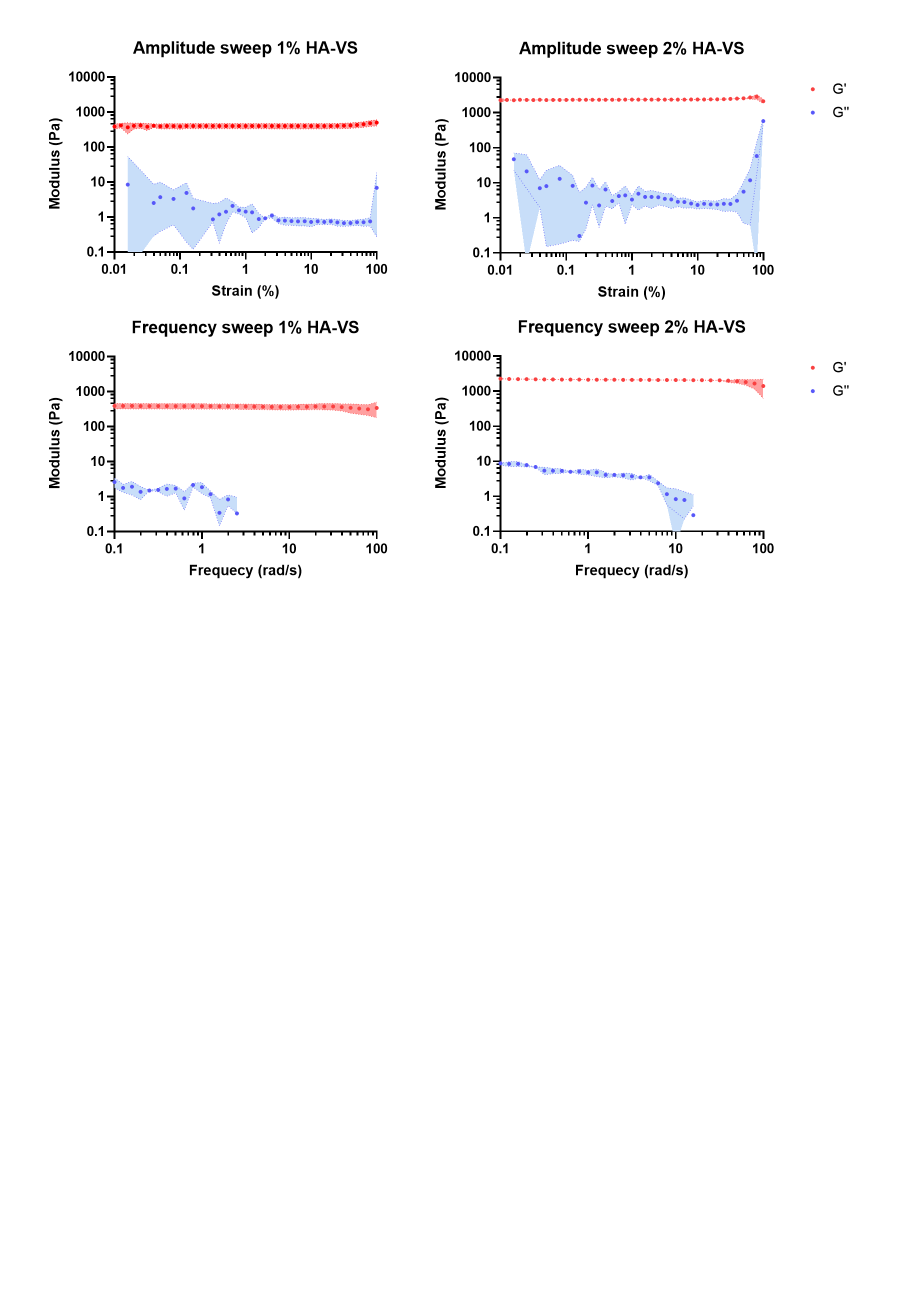


Supplementary figure 1. Mean amplitude and frequency sweep measurements of 1% and 2% HA-VS hydrogels showing the linear viscoelastic regime (*n* = 3 per sweep and HA-VS %, shaded area shows standard deviation).


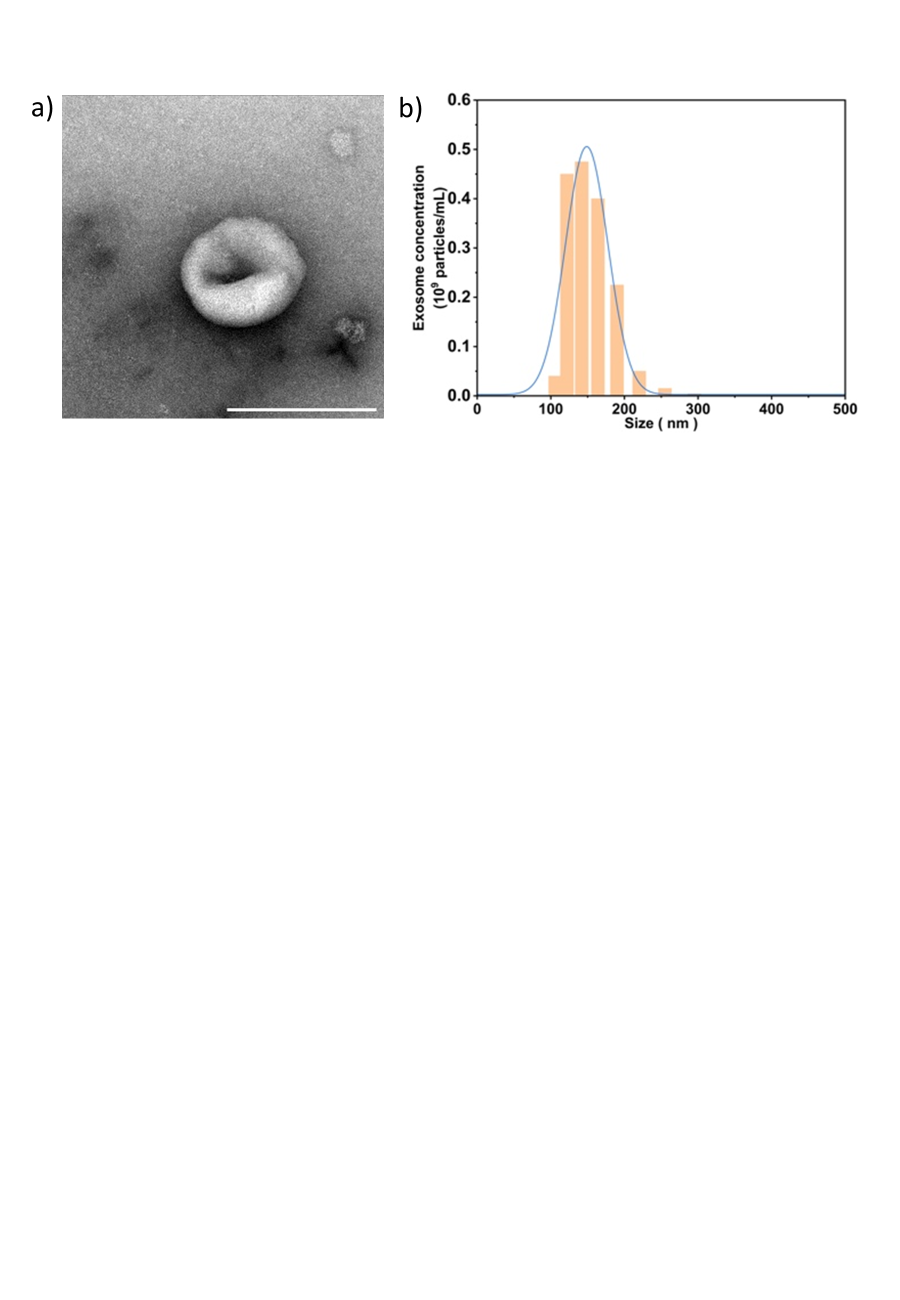


Supplementary figure 2. Characterization of exosomes derived from human dental pulp stem cells. a) Representative transmission electron microscopy (TEM) image of an exosome, confirming its typical cup-shaped morphology. Scale bar = 200 nm. b) Nanoparticle tracking analysis (NTA) data of the exosome population, showing the size distribution profile and concentration.
